# A Digital Platform for Home-Based Exercise Prescription for Older People with Sarcopenia

**DOI:** 10.3390/s24154788

**Published:** 2024-07-24

**Authors:** Matteo Bonato, Federica Marmondi, Claudio Mastropaolo, Cecilia Inzaghi, Camilla Cerizza, Laura Galli, Giuseppe Banfi, Paola Cinque

**Affiliations:** 1 Department of Biomedical Sciences for Health, Università degli Studi di Milano, Via Giuseppe Colombo 71, 20133 Milan, Italy; 2 Laboratory of Movement and Sport Sciences (LaMSS), IRCCS Istituto Ortopedico Galeazzi, Via Cristina da Belgioioso 173, 20157 Milan, Italy; cecilia.inzaghi@grupposandonato.it (C.I.); banfi.giuseppe@hsr.it (G.B.); 3 Unit of Infectious Diseases, IRCCS San Raffaele Scientific Institute, Via Stamira d’Ancona 20, 20127 Milan, Italy; marmondi.federica@hsr.it (F.M.); cerizza.camilla@hsr.it (C.C.); galli.laura@hsr.it (L.G.); cinque.paola@hsr.it (P.C.); 4 Visionage s.r.l., Via Guglielmo Marconi 19, 70011 Alberobello, Italy; claudio@visionage.it

**Keywords:** monitoring, health, smart devices, physical activity, exercise, sarcopenia, aging, active aging

## Abstract

Digital therapeutics refers to smartphone applications, software, and wearable devices that provide digital solutions to improve healthcare delivery. We developed a digital platform to support the GYM (Grow Your Muscle) study, an ongoing 48-week randomized, controlled trial on reduction of sarcopenia through a home-based, app-monitored physical exercise intervention. The GYM platform consists of a smartphone application including the exercise program and video tutorials of body-weight exercises, a wearable device to monitor heart rate during training, and a website for downloading training data to remotely monitor the exercise. The aim of this paper is to describe the platform in detail and to discuss the technical issues emerging during the study and those related to usability of the smartphone application through a retrospective survey. The main technical issue concerned the API level 33 upgrade, which did not enable participants using the Android operating systems to use the wearable device. The survey revealed some problems with viewing the video tutorials and with internet or smartphone connection. On the other hand, the smartphone application was reported to be easy to use and helpful to guide home exercising. Despite the issues encountered during the study, this digital-supported physical exercise intervention could provide useful to improve muscle measures of sarcopenia.

## 1. Introduction

People are living longer, and every country worldwide is growing in both size and proportion of older persons [1]. According to recent population projections, 1 in 6 people in the world will be aged 60 years or over by 2030. At that time the share of the world population aged 60 years and over will be increased from one billion in 2020 to 1.4 billion and will double by 2050 (2.1 billion). The number of persons aged 80 years or older is expected to triple between 2020 and 2050 to reach 426 million [2].

Aging results from the impact of the accumulation of a wide variety of molecular and cellular damage over time. This leads to a gradual decrease in physical and mental capacity, a growing risk of disease, and ultimately death [3]. Particularly, aging is associated with a decline in skeletal muscle mass and function, causing an alteration of strength and physical performance, which begins sooner in women and in persons with non-communicable diseases [4,5]. Weakness of lower extremity is not only associated with poor functional performance but also with higher risk of fall and disability [6]. Proprioceptive perception also decreases with age, and older people typically show a reduced balance control, resulting from both altered muscle function and sensory information, mainly through the somatosensory, vestibular, and visual systems [7]. Sarcopenia (Greek, Sarx for “flesh” and Penia for “loss”) defines the pathological condition associated with aging and characterized by the progressive loss of skeletal muscle mass, strength, and function [8,9] or, according to the Delphi Consensus from the global Leadership Initiative in Sarcopenia (GLIS), by the concurrent combination of reduced muscle mass and strength [10]. Sarcopenia is also recognized as an independent and pathological condition by an ICD-10CM code [11].

According to a recent systematic review with a metanalysis that included 263 studies, the prevalence of sarcopenia ranged between 0.2% and 86.6%, depending on study populations and definition criteria. Although the mainly used classifications are those from the European Working Group of Sarcopenia in Older People 2 (EWGSOP2) and the Asian Working Group on Sarcopenia (AWGS), many studies estimate sarcopenia solely by muscle mass adjusted for the squared of height [12]. The pathophysiology of sarcopenia is complex and results from biological alterations in muscle structure, hormonal imbalances, and external influences, for example, a reduced energy intake [13]. In addition, underweight people, women, and people with other chronic diseases are more likely to develop sarcopenia [12].

Sarcopenia has major adverse health outcomes, leading to a higher risk of functional disabilities, quality of life impairments and falls, and it is associated with a number of chronic conditions, including osteoporosis, dyslipidemia, increased cardiovascular risk, metabolic syndrome, and immunodepression [14]. Sarcopenia is also associated with a 50% increased risk of hospital admission, a 20-day increase in hospital stay length, and a 34% to 58% increase in hospital care cost [15].

To prevent and manage sarcopenia, evidence-based clinical practice guidelines provide strong recommendations for physical activity, with reference to resistance training exercises for improving skeletal muscle strength and mass [16]. However, no longitudinal intervention studies have to date specifically addressed the benefits of a physical activity intervention on sarcopenia as primary outcome.

In recent years, digital technologies gained popularity for physical and health monitoring, representing nowadays one of the most popular trends in health and fitness [17]. Digital therapeutics refers to evidence-based therapeutic interventions driven by high-quality software, wearable devices, internet-based programs, and smartphone applications [18]. They are used independently or in concert with medications or other approaches to optimize patient care and health outcomes [19], including physical activity programs. Digital therapeutics are becoming increasingly popular as a tool for exercise prescription, monitoring of daily physical activity and nutrition, and health-related parameters management. The use of digital devices not only may assist patients to pursue a healthier lifestyle but may also provide physiological and metabolic data that could be functional to the management of non-communicable diseases. Recent evidence suggests that the use of such technologies together with physical exercise interventions improve physical activity levels, physical fitness, body composition, and metabolic and psychological parameters, providing an important tool for developing physical activity programs to transfer to the clinical practice [20].

The GYM (Grow Your Muscle) study aims to validate a program of physical exercise for people with sarcopenia to reduce the burden of this disease in terms of quality of life and physical health. To this aim, we are using a digital platform developed specifically for this study, which consists of a smartphone application including the exercise program with video tutorials for the correct execution of home-based body-weight exercises, a wearable device to monitor heart rate (HR) during training, and a website for downloading training data that enable the GYM supervisors to remotely monitor the exercise. The aim of this paper is to describe the platform in detail and discuss the technical and practical issues related to its use in the participants of the experimental group who have so far been enrolled.

## 2. Materials and Methods

### 2.1. The GYM Study Design

The GYM study is a 48-week, parallel group, open-label, randomized, controlled, superiority trial comparing the effect of a physical activity intervention versus no exercise prescription in elderly persons from the general population (PGP) and persons with HIV (PWH) with sarcopenia. Inclusion criteria consist of being ≥60-year-old for PGP or ≥50-year-old for PWH, with sarcopenia, as defined by an Appendicular Skeletal Muscle Index (ASMMI) of ≤7.0 kg/m^2^ for men or ≤5.5 kg/m^2^ for women [21], or by Skeletal Muscle Index (SMI) of ≤10.75 kg/m^2^ for men or ≤6.75 kg/m^2^ for women by bioimpedentiometry (BIA 101 BIVA PRO, Akern s.r.l., Firenze, Italy) [22]. Exclusion criteria include any medical condition requiring hospitalization in the 6 weeks before enrollment or contraindicating physical exercise, as established by the sport medicine specialist (CC) and current substance and/or alcohol abuse. The study protocol has been approved by the San Raffaele Hospital Ethical Committee (197/INT/2020), in accordance with current national and international laws and regulations governing the use of human subjects (Declaration of Helsinki III). This trial is registered at ClinicalTrials.gov (NCT05071040).

The primary objective is to assess the effectiveness of a home-based, body-weight resistance training protocol delivered by the digital GYM platform to improve muscle strength, as by handgrip strength, in PGP and PWH, at the short (W12) and longer term (W48). Secondary objectives are to assess W12 and W48 changes from BL of body composition, physical tests, blood lipid profile, cognitive and psychological scores, muscle measures by magnetic resonance imaging, and blood biomarkers of inflammation and muscle health.

### 2.2. Participant Screening and Assessments

Potential participants are screened for sarcopenia by BIA, and those meeting the GYM inclusion criteria are invited to participate to the study. Baseline (BL) evaluations are carried out on two different days. On the first day, evaluations are performed at the outpatient clinic of the IRCCS San Raffaele Turro Hospital (Milan, Italy) and consist of a medical examination by the sport medicine specialist (CC), through the collection of clinical and treatment history, anthropometric assessment, physical examination, and electrocardiogram at rest. Participants with no contraindication to perform physical exercise are enrolled and perform physical tests and cognitive and psychological assessments, blood sampling on the same day.

Physical tests include the handgrip strength [23], maximal isometric strength of knee extensor muscles [5], chair-stand test [24], the Mini Balance Evaluation System (Mini-BESTest) [25], and the six-minute walking test (6MWT) [26]. Cognitive function is assessed by Trail Making test A (TMA), Trail Making Test B (TMB) [27], and digit symbol [28], whereas psychological and quality-of-life assessments are performed through the validated version questionnaire of the Profile of Mood States (POMS) [29] and of Quality of life SF-12 health survey [30].

Blood is drawn for standard examination and collection of blood cells and plasma for subsequent biomarker assessment. Standard exams include blood cell count, fasting total, HDL and LDL cholesterol, triglycerides, glucose, insulin, glycated hemoglobin (HbA1c), creatinine, ALT, and AST. CD4+ and CD8+ T-cell counts, HIV-1-RNA plasma level, and the Veteran Aging Cohort Study Risk (VACS) index [31] are also evaluated in PWH. Biomarkers will include soluble and cell markers of inflammation and muscle health.

Participants are instructed to present at the first visit in a fully hydrated state, taking their medication as usual, at least 8 h after the last meal, and to avoid exercise in the 24 h preceding the visit. Because participants generally have no experience with physical testing, they are familiarized in advance with all procedures.

On the second day, approximately two weeks after the initial visit, participants perform radiological assessments at the IRCCS Ospedale Galeazzi-Sant’Ambrogio (Milan, Italy). These consist of a total body composition study by Dual-Energy X-ray Absorptiometry (Hologic QDR-Discovery Densitometer; Hologic Inc., Bedford, MA, USA) to assess total fat mass and fat-free mass at arms, limbs, trunk, total body and ASMMI, and a magnetic resonance imaging (MRI) scan to objectively evaluate cross-sectional area and intermuscular adipose tissue of the thigh. MRI is performed on Dixon sequences on the axial plane at the middle third of the thigh using a 1.5T MR system (Avanto, Siemens Medical Solution, Erlangen, Germany).

Following the baseline (BL) evaluations, participants are randomized with a 1:1 ratio, by a computer-generated blocked randomization list, to either the experimental group (EG), where they receive the exercise prescription delivered by the GYM platform, or to the non-exercise control group (CG). Participants of both groups receive a booklet containing general advice on the benefits of physical activity following the recommendations of the American College of Sport Medicine [32].

All the participants are also evaluated at W12 for all the BL tests except for the radiological examination and at W48 for the whole panel of BL tests.

### 2.3. The GYM Home-Based Body-Weight Training Protocol

Following randomization, the EG participants are instructed and familiarized with the correct execution of the exercises during a single supervised training session by a formed investigator (CI).

The GYM protocol consists of four home-based sessions per week, for 48 consecutive weeks (192 total training sessions), of body-weight exercises, including 7 exercises of light joint mobility for warm-up (5 min), 8 body-weight resistance training exercises (20 min), and 5 strength exercises for cooldown (5 min). Warm-up includes light dynamic movement and exercises for upper and lower limbs, neck, and back. The resistance training part consists of exercises with only body-weight exercises or a few extra weights when needed with small (approximately 500 g) or large (approximately 1500 g) water bottles, plus a monopodalic balance exercise. Table 1 illustrates the details of the central part resistance training exercise, with number of sets, repetitions, resting periods, and exercise progression. The cooldown consists of light static stretch exercises, lasting between 25 and 40 sec for the main body muscles previously involved during the training session, including neck, back, and upper and lower limbs.

Recommendations are given for exercise progression. During week 0 to week 6: participants start with the initially suggested numbers of sets and repetitions, based on the degree of the participant’s training phase; week 7–12: increase the number of repetitions, but not the number of sets; week 13–18: maintain the number of repetitions, but increase the number of sets; week 19–24: maintain the number of sets and repetitions and, when possible, modify the exercise execution modality of calf, squat, and push-ups or use a larger bottle of water as extra weight for lateral raises, biceps and triceps curl; week 25–30: maintain the number of sets and repetitions and the new exercise modalities; week 31–36: increase the number of repetitions, but not the number of sets with the new exercise modality; week 37–42: maintain the number of repetitions, but increase the number of sets with the new exercise modality; week 43–48: maintain the number of sets and repetitions and modify again the exercise modality of calf, squat, push-ups or using a larger bottle of water as extra weight (lateral raises, biceps and triceps curl).

### 2.4. The GYM Platform

The GYM Platform was developed specifically for this study and consists of a smartphone application, a wearable device, and a dedicated website. The version used in this study is in Italian.

**Figure 1:**
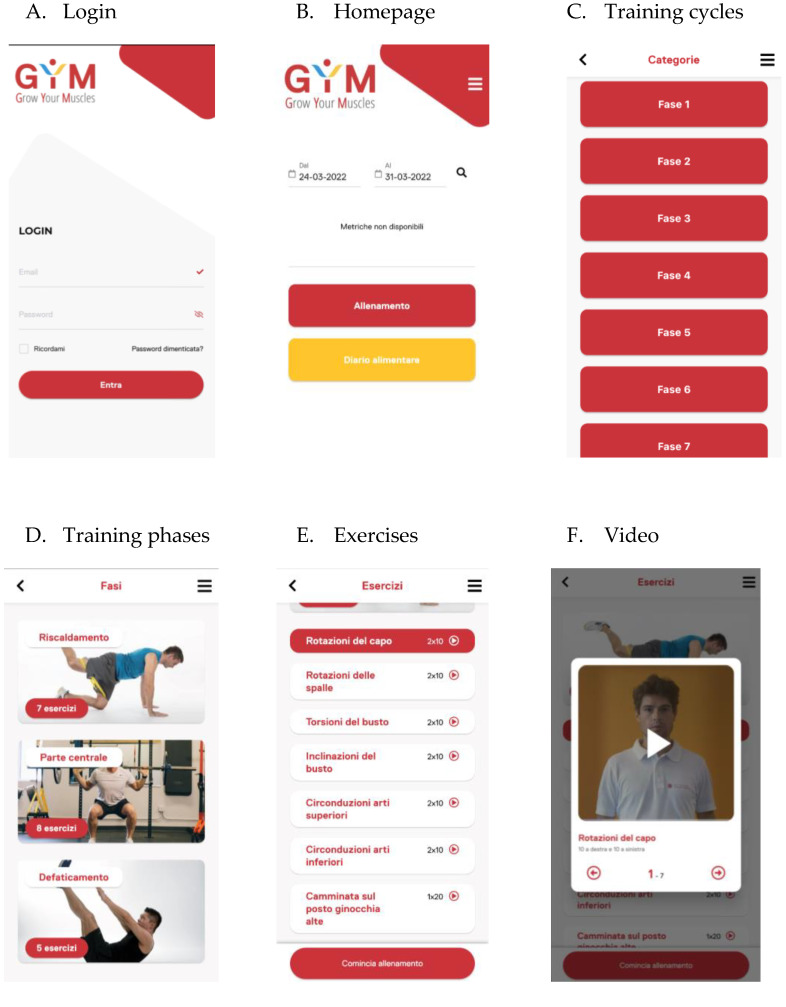
The GYM (Grow Your Muscles) smartphone application.

The smartphone application is available for EG study participants for iOS and Android by entering in the research field “Gym–Grow Your Muscles” or using the link https://visionage.it/gym.html. Participants download the application on their personal smartphone and log in with a personal username and password every time they use the GYM application (Figure 1A). Once logged in, they can check their metrics about training, start a new training session by clicking the red button (‘Allenamento’), or consult the general advice on the benefits of physical activity, also including dietary advice, by clicking the yellow button (‘Diario alimentare’) (Figure 1B). Before starting the training session, participants are requested to choose the correct training cycle, according to the week of protocol (Figure 1C). They then start with the warm-up (‘Riscaldamento’), continue with the body-weight resistance exercises (‘Parte centrale’), and finally go to the cooldown exercises (‘Defaticamento’) (Figure 1D). Participants are required to complete the program by clicking the exercise (Figure 1E) and can follow the video tutorial (Figure 1F). The total training time is registered and sent to the GYM website at the end of each training session. To properly use the GYM application, participants need a fast internet connection, possibly through a personal wireless network, the Bluetooth connection turned on for the heart band connection, not to hold the smartphone in energy-saving mode, and to keep the smartphone’s operating system always updated.

**Figure 2:**
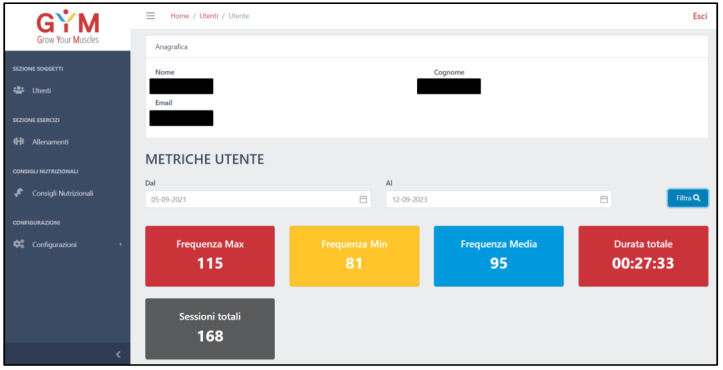
The GYM (Grow Your Muscle) Website.

During the 48 weeks of the study, the participants randomized to the EG are equipped with a wearable device consisting of a heart rate (HR) band (Polar H9, Polar Electro 2011, Kempele, Finland), which is connected via Bluetooth to the participant’s smartphone. Participants are instructed to wear the HR band at the chest prior to the start of each training session to measure maximal, minimum, and average HR, which will be sent to the GYM website at the end of each training session. Through the GYM website (https://gym.visionage.it/#/login), the study supervisors (MB, FM) can remotely monitor adherence for each EG participant, by recording the number of training sessions performed, the training time of each session, and the effective training protocol execution, by recording the maximal, minimum, and average HR (Figure 2).

Following randomization, and after the session of familiarization with execution of the exercises, the EG participants are also instructed on and familiarized with the correct use of the GYM application and of wearing the HR band. During the 48 weeks of the study, a person from our team (FM) is dedicated to help participants regarding specific requests for advice or problem-solving by phone counselling or in person.

### 2.5. Usability of the Smartphone GYM Application

To assess the usability of the smartphone GYM application, we carried out a short survey that was created on Google Forms and sent to all EG participants. The survey was delivered in Italian and took 2–5 min to complete. It consisted of 8 different items:

(A) Gender: male, female, other.
(B) Age: years old.
(C) Study start date: year and the month of the recruitment visit.
(D) Smartphone operating system: Android or Apple (iOS).
(E) Utility of the GYM application to carry-out the home-based body-weight exercises:
  - Not useful
  - Not very useful
  - Quite useful
  - Very useful
  - Fundamental
  - Not evaluable
(F) Indication of one or more positive aspects relating to the use of the GYM application:
  - It allowed me to perform physical activity at home.
  - The video helped me to do the exercises correctly.
  - It motivated me to perform the exercises regularly.
  - It allowed me to organize the physical activity based on my daily commitments.
  - It is easy to use.
(G) Indication of one or more negative aspects relating to the use of the GYM application:
  - Problems with internet connection
  - Problems with access and/or operating system
  - Problems in viewing the exercise videos
  - Difficulty in reading the text
  - Difficulty in using the HR band
(H) Any additional comment

### 2.6. Descriptive Statistical Analysis

The usefulness and the positive and negative practical issues of the GYM application were described based on the answers gathered from the survey. Descriptive statistics used median and 25th–75th interquartile (IQR) values and was performed using GraphPad Prism software, version 11 for Windows (GraphPad Software, San Diego, CA, USA).

## 3. Results

### 3.1. Current Status of Study Enrolment and Adherence to the Protocol

One hundred and ninety-six subjects are planned to participate to the study. Of these, 157 (80%) have been enrolled so far: 28/157 (18%) have dropped out during the study, 38 have completed the W48 study and 91 are still on study. Overall, 64 EG participants have used the GYM application.

**Figure 3:**
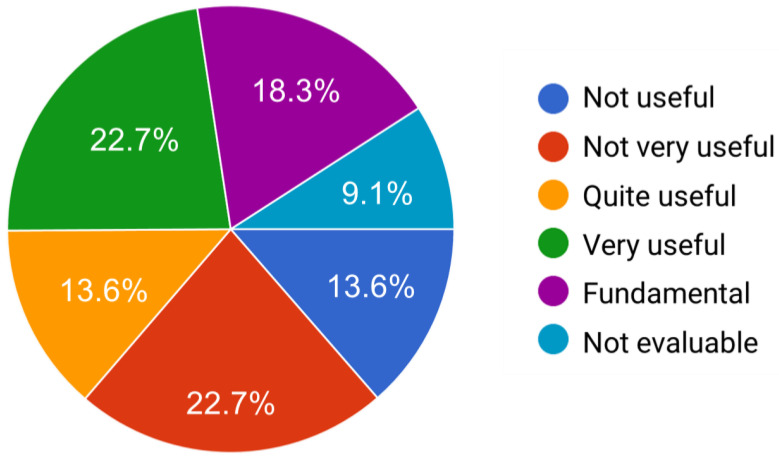
Usefulness of GYM application to carry out home-based body-weight exercises, as reported by the participants of the Experimental Group.

The median training adherence during the 48-week training protocol was assessed in the 38 EG participants who completed the study through the data collected in the GYM website, as described in the Methods section and illustrated in Figure 3. Median adherence was 83% (IQR 40–93%), and for each participant the output data on HR mean, HR min, HR max, and training time were consistent with the expected exercise intensity and duration based on the input program.

### 3.2. Technical Issues Related to the Use of the GYM Platform

We encountered one main technical issue related to the GYM application, which presented with the API level 33 update (released on 1 November 2023) for the Google Play Console. Following the upgrade, participants using Android could no longer find the GYM application on the Google Play Store. To solve this problem, we updated the user experience, security, and performance of the Android platform, thus enabling Android participants to download the GYM application by sending a link or a QR code. While waiting for the issue to be fixed, the participants using Android received a hard copy training program together with a diary, to register the time and duration of the training sessions.

However, a more severe consequence of the API level 33 update concerned the connection of the Polar H9 device to the HR band, because Polar did not update the API of their devices to the new Google-imposed standards. We had neither the time nor the resources to solve this issue, and therefore, since the time of the update, Android users had to exercise without wearing the HR band and we only monitored time and duration of the training sessions through self-reported data in a diary.

### 3.3. Usability of the GYM Smartphone Application—Survey Results

Out of 64 EG participants enrolled so far, 49 (76%) completed the survey. They were 29 (60%) men and 20 (40%) women, median age was 66 years (IQR, 63–70), with a median time of participation in the study of 6 months (IQR, 3–11). The smartphone operating system was Android in 27 of 49 participants (55%) and Apple iOS in 22 of 49 (45%), and none of them switched to the other operating system during the study.

Figure 3 shows the survey results on the usefulness of the GYM application to carry out the home-based body-weight exercises. More than half of the EG participants (54.5%) reported that it was fundamental, very useful, or quite useful, whereas 36.3% found it not useful or not very useful. Its usefulness was not evaluated by 9.1% of the participants in the survey.

**Figure 4:**
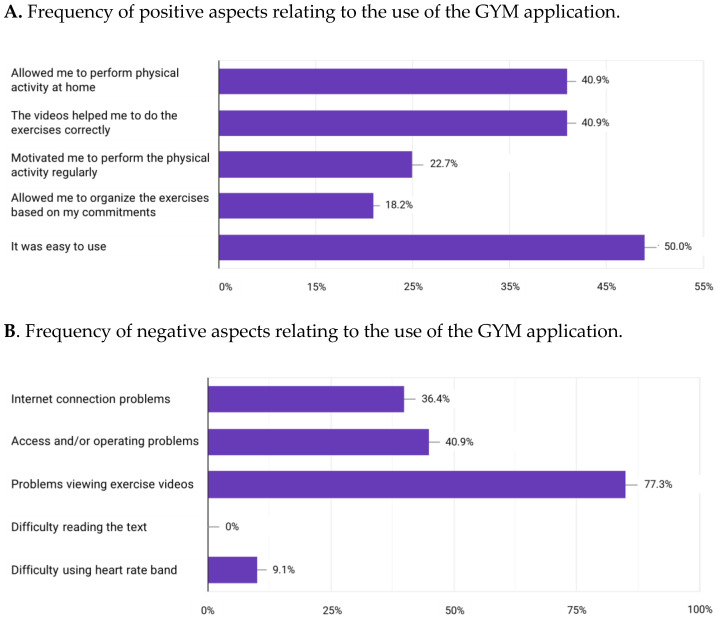
Frequency of positive (**A**) and negative (**B**) aspects relating to the use of the GYM application, as reported by the participants of the Experimental Group.

Figure 4 shows the survey results on the positive (Figure 4A) and negative (Figure 4B) aspects of the use of the GYM application. The most frequently reported positive aspect was the easiness of use (50%), whereas the most frequently reported negative aspect (77.3%) was technical and concerned problems at viewing the exercise videos.

## 4. Discussion

We describe here an integrated platform for digital therapeutics consisting of a smartphone application, a wearable device, and a website, for prescribing and monitoring a physical exercise intervention for elderly people with a specific medical condition. This platform is a key tool in the GYM study, an ongoing 48-week randomized, controlled trial on reduction of sarcopenia through a home-based, app-monitored physical exercise program.

The beneficial effects of physical activity for prevention and management of sarcopenia are well documented [33]. We designed the GYM platform specifically to help elderly people with sarcopenia perform a home-based physical activity. Exercising at home may have some practical advantages compared to exercising outdoors, especially in older persons at higher risk and fear of fall, due to low muscle mass and strength and frailty. More in general, home exercising is easy and convenient to perform, time- and cost-saving, and enables flexibility to exercise at any time of the day in a comfortable environment. In the GYM protocol, each session lasts 30–40 min, which is relatively short and well suited to a session of home exercise, and video tutorials are provided in the GYM application as a guidance with the body-weight exercises and to perform exercises correctly.

In addition, the GYM platform allows for monitoring a high number of participants at the same time. In fact, once the participant ends the training session, the data relating to its duration and HR are sent to the website and made available to the GYM supervisors, who can monitor the activity in real time. Remote training monitoring may be useful to increase motivation to exercise and thus improve adherence to the program. In addition, it enables supervisors to help solve problems with exercise performance or adherence. In our study, if a participant misses several sessions in a row, the team calls to ensure that there are no relevant problems, e.g., regarding exercise prescription, health problems, or issues relating to the correct functioning of the GYM platform. Indeed, it is well known that structured, supervised, home-based exercise programs can increase exercise behaviors in older adults [32] and that supervision by an experienced fitness professional can enhance physical activity adherence [34].

However, during our trial we were dealing with a major technical issue, which was related to the upgrade of the Android operating system to the API level 33. While we were able to fix the access problems to the GYM application resulting from the upgrade, this upgrade prevented the use of the Polar H9 health band, because Polar did not update the API of their devices to the new standards. As a result, HR measurements during the training sessions and their duration were no longer feasible since the date of the upgrade in approximately half of our EG participants, i.e., all the Android users. We managed to record adherence by asking participants to self-report the training time in a diary, but the HR information was not recorded. More in general, these issues highlight some relevant concerns that may arise due to the rapid technology evolution, with a great variety of smartphones and operating systems that could affect the use of specific exercise applications, and even more so when connections with other devices are involved.

The usability level as well as the challenges of the GYM application were further investigated through the retrospective survey. The main issue encountered by EG participants included problems with viewing the video tutorials. It is key that such issues be promptly solved, because they may affect substantially both adherences to exercise prescription and proper training, which may in turn reduce the efficacy of the intervention. Indeed, as already observed [35,36], participants might end up giving more attention to the device functioning than to the exercise activity, thus reducing their motivation to exercise. Other technical issues reported through the survey may have depended on low users’ digital literacy. Indeed, older people may not always be skilled for interaction with technology-based health tools, which may have affected the interaction with the GYM platform. Participants had to log in to the GYM application by using a personal username and password, but sometimes credentials were not saved or lost following application logout. Sometimes, participants also happened to accidentally disconnect the app from the health band and were then unable to reconnect. We tried to reduce the impact of these issues by dedicating a person from our team to help participants by phone counselling or by inviting them to the clinic to solve the problem in person.

On the other hand, the questionnaire also provided encouraging feedback. Overall, the GYM platform was regarded as useful by most of the participants, with the most frequently reported positive aspects being its easiness of use, as well as the help provided to perform the exercises correctly and using the video tutorials. However, it must be underlined that, beyond the technical positive aspects reported, there was also a positive participants’ attitude, likely resulting from the clearly defined health goals, expected to be achieved specifically through the home-based training protocol. Indeed, at the time of writing, the drop-out rate from the study was 18%, which is like that observed in other physical exercise studies of shorter duration [37].

Digital therapeutics represents a promising evolution in healthcare, offering innovative solutions that may complement traditional approaches. Introducing digital platforms for physical exercise prescription in the clinical practice is challenging, but it would have several advantages, including the potential to improve accessibility of older people to physical activity, with all the health benefits. To achieve these goals, however, digital platforms for exercise prescription and monitoring need to be tested in longitudinal intervention studies [38]. We believe that our study provides information that may be useful in this perspective and for the design of future studies.

In addition, the rapid evolution of the digital technologies offers additional tools that may improve usability and efficacy of digital platforms for health and could also be considered for future development of the GYM platform. These include, among others, a possible multimodal usage of the platform, its access through a biometric login system, or its integration with artificial intelligence and machine learning approaches. A multimodal usage of the GYM platform might allow participants to receive their exercise prescription in the most convenient format according to their needs and by integrating the access across a wide range of devices, thus facilitating exercising in different contexts and situations. The access to the platform by biometric login systems, such as fingerprint, facial, or voice recognition, would eliminate the need for traditional passwords, providing a more rapid and friendly authentication to the smartphone application. Artificial intelligence and machine learning approaches may help, at the same time, to standardize and improve the quality of prescription and monitoring of exercise, through the use of algorithms including individual health status, needs, and limitations among the relevant information. These approaches may also be helpful to modify exercise protocols over time based on data output, i.e., adherence and correct exercise performance. 

## 5. Conclusions

In conclusion, the maintenance of the functional muscle capacity is a primary goal in the management of elderly people with sarcopenia. We expect that our physical activity program, supported using the ad hoc GYM platform, will prove to be useful to improve measures of sarcopenia and thus reduce the consequences of this condition.

The implementation of digital therapeutics may be key to prescribing and monitoring physical activity and, in this perspective, the GYM platform may represent an example and a step towards integration of digital devices in the clinical practice. An important consideration, however, relates to the continuous rapid technological evolution and the consequent need to update digital therapeutic tools to the new standards in real time. The implementation of integrated systems that could be easily used as digital therapeutics would require an improved connection between healthcare organizations and digital companies. Accordingly, the establishment of multidisciplinary teams including both healthcare providers and information technology experts would be essential to transfer simple and easy-to-use medical tools into clinical practice.

## Figures and Tables

**Table 1:** Description of the resistance training exercises included in the central part of the home-based training protocol.

Exercise					Progression
Glute extension	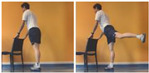				Start from 3 sets for 10 repetitions and increase up to 4 sets and up to 20 repetitions.
Calf extension	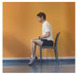	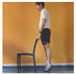		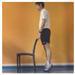	Start from 3 sets for 10 repetitions and increase up to 4 sets and up to 20 repetitions and/or changingcalf exercise.
	Sitting	Standing		Monopodalic	
Squat	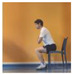		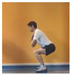		Start from 3 sets for 10 repetitions and increase up to 4 sets and up to 20 repetitions and/or changing squat exercise.
	With chair		Free		
Monopodalic balance	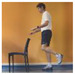				Start from 3 sets of 10 s and increase up to 4 sets and up to 1 min.
Push-ups	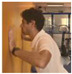		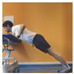		Start from 3 sets for 10 repetitions and increase up to 20 repetitions and/or changing push-up exercise.
	Against wall		At table		
Lateral raises	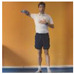				Start from 3 sets for 10 repetitions and increase up to 4 sets and up to 20 repetitions and/or changing weight bottle.
Biceps curl	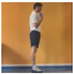				Start from 3 sets for 10 repetitions and increase up to 4 sets and up to 20 repetitions and/or changing weight bottle.
Triceps curl	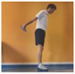				Start from 3 sets for 10 repetitions and increase up to 4 sets and up to 20 repetitions and/or changing weight bottle.

## Data Availability

The data that support the findings of this study are available from the corresponding author upon reasonable request.
